# Superior mesenteric artery syndrome: when vomiting are not voluntary

**DOI:** 10.1192/j.eurpsy.2023.1803

**Published:** 2023-07-19

**Authors:** M. A. Morillas Romerosa, A. Oliva Lozano, P. Herrero Ortega, J. Garde Gonzalez

**Affiliations:** Psiquiatría, Hospital Universitario la Paz, Madrid, Spain

## Abstract

**Introduction:**

Superior mesenteric artery syndrome is a gastro-vascular disorder in which the third and final portion of the duodenum is compressed between the abdominal aorta and the overlying superior mesenteric artery. This rare, potentially life-threatening syndrome is typically caused by an angle of 6°–25° between the abdominal aorta and the superior mesenteric artery, in comparison to the normal range of 38°–56°, due to a lack of retroperitoneal and visceral fat (mesenteric fat). In addition, the aortomesenteric distance is 2–8 millimeters, as opposed to the typical 10–20. However, a narrow superior mesenteric artery angle alone is not enough to make a diagnosis with no symptoms.

Symptoms are fullness and epigastric tightness after meals, nausea and vomiting (often bilious) and pain in the middle of the abdomen that improves with the prone or knees flexed to the chest. The diagnosis is supported by imaging tests (esophagogastroduodenal transit or CT) showing dilation and stasis proximal to AMS in the third duodenal portion.

Relief from vomiting with feeding through a enteral probe placed beyond the obstruction to the proximal jejunum supports diagnosis.

Precipitating factors should be corrected first, whenever possible. Acute symptoms can be resolved with gastric decompression and intravenous fluids. Therefore, surgical correction should only be done in well-studied patients with chronic recurrent episodes of AMS syndrome. The most recommended surgical technique is a laparoscopic proximal duodenojejunostomy

**Objectives:**

To describe a case of superior mesenteric artery syndrome and review in literature the organic complications and associated psychopathology of this disorder

**Methods:**

Clinical case report and brief review of literatura

**Results:**

17-year-old woman with a diagnosis of anorexia nervosa. Admitted for behavioral disorder, repeated self-harm and low mood. Presents a BMI of 16.6. Irregular rules. Progressive diet is started to which nutritional supplements are added with good initial tolerance. It presents a loss of 2kg and begins with nausea, vomiting and postprandial epigastralgia. Oral panendoscopy and abdominal ultrasound are performed showing possible mesenteric aortic clamp so naso-jejunal probe and exclusive enteral feeding is prescribed. She received enteral jejunal nutrition progressively with feedback syndrome prophylaxis that included parenteral vitamin B1. After a few days, oral supplementation began. He remained hemodynamically stable, with no signs of heart failure. It gained 3kg of weight up to 43.2kg, starting before discharge from the hospital successfully oral tolerance.

**Conclusions:**

Superior mesenteric artery syndrome is a serious complication in anorexia nervosa with a low incidence and an estimated mortality of 33%. A multidisplinar approach that addresses both the medical and psychological needs of these patients throughout their hospital stay is necessary.

**Disclosure of Interest:**

None Declared

